# Development of Measurement Equipment and Experimental and Numerical Simulation Studies for Warm Forming Limits of High-Strength Steel

**DOI:** 10.3390/ma14092373

**Published:** 2021-05-02

**Authors:** Qiang Yu, Jin Liang, Qiu Li, Chengyao Li

**Affiliations:** 1 State Key Laboratory for Manufacturing Systems Engineering, School of Mechanical Engineering, Xi’an Jiaotong University, Xi’an 710049, China; liangjin@mail.xjtu.edu.cn (J.L.); CY_leo@yeah.net (C.L.); 2 School of Mechanical Engineering, Tianjin University of Technology and Education, Tianjin 300222, China; liqiu@tute.edu.cn

**Keywords:** warm forming, forming limits, high-strength steel

## Abstract

This paper describes the research and development of a set of measurement equipment for the warm forming limits of high-strength steel based on the Nakazima bulging test method and the digital image correlation method. The equipment could provide an argon shield and a water-cooling atmosphere, as well as two heating options: heating the specimen, dies, and environment to the test temperature simultaneously or heating the specimen to the test temperature at a higher speed than that for the dies and environment. The equipment was applied to measure the forming limit curves for high-strength DP600 steel at room temperature and at the temperature of 300 °C to verify its performance. The DYNAFORM software was then applied for the digital simulation of the bulging test method. A new limit-strain-fitting method was proposed to eliminate the impact of the distorted grid on the digital simulation process. The change trend of the forming limit curve acquired in the test had sound consistency with the test results.

## 1. Introduction

Energy conservation and emission reduction are constant pursuits of the automobile industry. Reducing the weight of a vehicle’s body without it losing its strength is an important approach for improving the fuel efficiency and reducing emissions. Among the materials used for manufacturing car bodies, high-strength steel plates are widely applied for their high specific strength. In the stamping of structures for the vehicle bodies, however, high-strength steel may lead to the formation of defects, such as necking and cracks, resulting in difficulties in the formation under normal temperatures [1]. On account of its performance in reducing the transformation resistance and overcoming the defects from the difficulties of processing high-strength steel under normal temperatures, warm forming (the forming temperature range is from room temperature to the recrystallization temperature of the material) is a preferable solution for forming parts from high-strength steel sheets [2,3]. Therefore, the forming performance of high-strength steel under warm conditions should be thoroughly understood; the performance is the precondition for the design and warm-forming manufacturing of the structures of high-strength-steel vehicle bodies.

The forming limit curve (FLC), which is plotted from the limits under different strain paths, is one of the most popular criteria for measuring the forming performance of sheet materials [4]. The FLC could be used to resolve the problems of process optimization and material selection in the processing of sheet materials [5,6]. The forming limit curve can be acquired via experimental measurement approaches, numerical simulations, and computation methods. Much research about experimental measurement of the FLC has already been performed, and the main forming methods applied in this research included the Nakazima hemispherical die bulging method [7], Marciniak flat punch steel die bulging method [8], cross-shaped dual-direction stretching method [9], etc. The research about the experimental measurement of the FLC for sheet materials under warm conditions also achieved satisfactory progress. Liu et al. [10] established the forming limit curve for high-strength DP780 steel at the typical temperature points from room temperature to 500 °C. Pandre et al. [11] performed an investigation of processing temperatures (room temperature, 200 °C, and 400 °C) on the forming limit diagram (FLD) of a thin-rolled DP590 sheet. In addition, the warm-forming limits of an aluminum alloy under certain temperature conditions were also found [12,13]. In the above experimental studies on the warm-forming limits of sheet materials, the limit strain was mainly obtained and measured via the grid strain method. In this method, grids are printed on the surface of a specimen through chemical corrosion, electrochemical corrosion, laser etching, or other techniques, and the limit strain is determined according to the deformation of the grid before and after the forming process. This method is applicable for the direct measurement of positions printed with grids, while the measurement accuracy for the limit strain incurred in positions beyond the grids cannot be guaranteed with this method. The digital image correlation (DIC) method is a non-contact measurement method that could cover all positions [14,15]. Our research team already established a strain measurement system for the prediction of forming limits at room temperature based on DIC, and we verified the performance of the system in the measurement of forming limits at room temperature through a comparison with the results from the traditional measurement of grid printing [16]. On this basis, this paper integrated the digital image measurement technique and a bulging test for sheet materials in a warm environment and designed and manufactured a set of measurement equipment for the warm-forming limits of high-strength steel sheets. The equipment was able to achieve full-field strain distribution of the deformed sheet in the full process of warm forming and normal temperature forming of high-strength steel. The equipment was applied to measure the forming limit curves for high-strength DP600 steel at room temperature and at the temperature of 300 °C to verify its performance.

The FLD obtained from the experiments was recognized as the most effective and accurate method for determining the formability of materials. However, the acquisition of the FLD for sheets with different materials and forming parameters would require a large number of experiments, which may need a great input in terms of cost and time. On account of its advantages, such as high computing speed and low cost, the numerical simulation computing method is another important technique in the prediction of forming limits. Lumelskyj et al. [17] used explicit dynamic analysis software to conduct numerical simulations of the Nakazima experimental process for steel and obtained prediction results for the forming limits. Bandyopadhyay et al. [18] adopted the LS-DYNA 971 numerical simulation software and used the modified Marciniak–Kuckzinki (MK) forming limit diagram acquisition method to predict relative parameters of the forming limits. Behrens et al. [19] adopted the ABAQUS finite element software to carry out numerical simulation calculations for the bulging process and obtained the FLD at room temperature for high-strength 7050 aluminum alloy and the FLD at a warm temperature for the AZ31 magnesium alloy. In addition to the above software, the DYNAFORM software has also been widely applied in numerical simulations of the forming process of sheet materials due to its high computing efficiency and the absence of requirements for sub-program setting of material parameters. In the simulation process of the DYNAFORM software, when the material reaches the forming limit, however, the connection between grids cannot be broken, leading to inaccuracy in the computing results for the forming limits. In response to this problem, this paper put forward a limit-strain-fitting method to overcome the distortion of the grid, which can rapidly and accurately obtain the forming limits under multiple temperature conditions by taking advantage of the forming results in numerical simulations.

## 2. Design of Warm-Forming-Limit Measurement Equipment

The warm-forming-limit measurement equipment for sheet materials was designed on the basis of the Nakazima experimental method and relevant technologies for digital image correlation. The equipment mainly consists of two parts: a part for warm forming and a part for digital image measurement. The design purpose of the equipment is to achieve two functions: automatic stamping and forming of sheet materials under warm conditions from the beginning of the experiment until the rupture of the sheet metal; image collection for the surface status of sheet materials in the warm-forming process, as well as the extraction of the strain under limiting status.

### 2.1. Structural Design of the Part for Warm Forming

**Figure 1:**
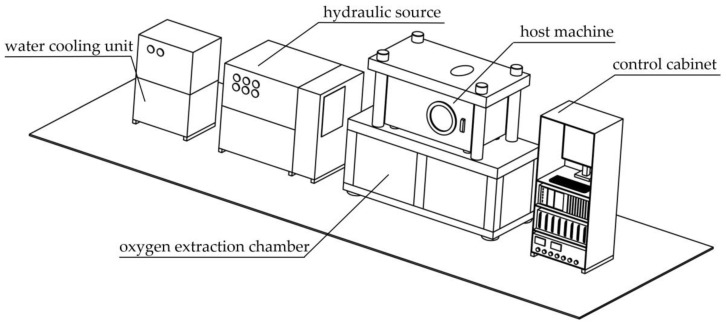
The forming part in the warm-forming-limit measurement system.

As shown in Figure 1, the forming part of the warm-forming-limit measurement system is constituted by the host machine, hydraulic source, water-cooling unit, oxygen extraction chamber, control cabinet, and relevant pipelines.

**Figure 2:**
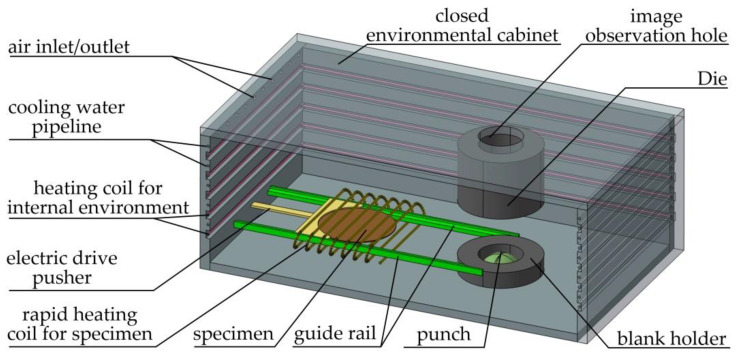
Schematic diagram of host structure.

As shown in Figure 2, the structure of the host machine consisted of a closed environmental cabinet, punch, die, blank holder, heating coil for the internal environment of the cabinet, rapid heating coil for the specimen, electric drive pusher, guide rail, etc. Of these, the die adopted an open structure. The image observation hole at the upper position of the closed environmental cabinet was installed with a temperature-resistant quartz glass with a thickness of 8 mm for the purpose of thermal insulation and transparency, which would facilitate the image collection of the camera in the upper positions in the forming process of the sheet material.

The punch, die, blank holder, and electric drive pusher were made of hot-work die steel, the guide rail was made of temperature-resistant ceramic material, and the four walls in the cabinet body were treated with asbestos boards for insulation and thermal preservation purposes. A heating coil for the space environment and thermocouples were applied on three walls for the environmental heating and temperature control of the space within the cabinet body. The temperature of the specimen and the ambient temperature in the cabinet were measured with thermocouples. Thermocouples were embedded in the guide rail and blank holder to determine the temperature of the specimen. The temperature of the specimen and inside the closed environment cabinet were obtained and displayed on a real-time basis through a programmable logic controller (PLC). A temperature-resistant quartz glass with a thickness of 8 mm was embedded into the front opening of the cabinet body to facilitate the observation of the experimental process. The die was fixed on the upper position of the forming cabinet, and the movements of the punch and blank holder at the lower position were under the accurate control of the hydraulic system with the assistance of a position sensor and a force sensor. The cabinet collected data such as displacement, force, and so forth to display the stamping force, cupping test values, and other information from the material stamping process. Once the stamping force decreased by 5% due to cracks in the specimen, the stamping process would stop automatically, and the punch and blank holder would withdraw back to their original positions.

The oxygen extraction chamber was connected to the host by a pipeline, and the argon gas inlet was used for oxygen extraction and the injection of argon gas for the removal of oxygen. The device was designed to guarantee an oxygen-free environment within the furnace to prevent oxidation reactions in the specimen under high temperatures. Dynamic sealing was adopted between the punch, blank holder, and host machine box to guarantee that the oxygen-free environment would have no changes in the host machine during the working process. The self-circulation of the water-cooling unit could guarantee the free flow of water in the cooling channel to rapidly cool the specimen and die or to adjust the internal temperature of the environmental chamber.

The main performance parameters of the warm-forming part are shown in Table 1.

### 2.2. Design of the Digital Image Measurement Part

**Figure 3:**
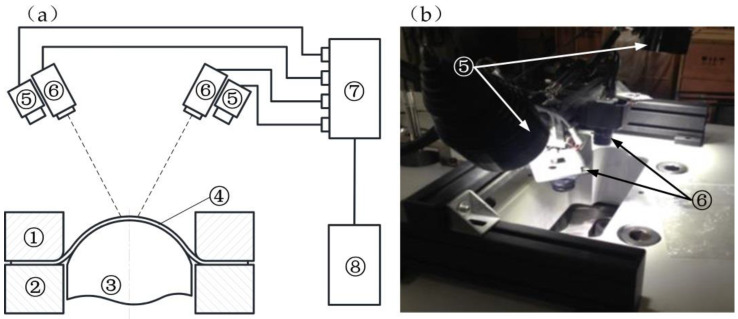
Structure of the digital image measurement part. (**a**) Schematic diagram; (**b**) physical picture. Components labeled in the figure are as follows: ① die; ② blank holder; ③ punch; ④ specimen; ⑤ LED light source; ⑥ camera; ⑦ controller; ⑧ computer.

The structure of the digital image measurement part is shown in Figure 3a. Two high-speed cameras were placed above the image observation hole of the host. The adopted cameras were MV-VS120FM black and white digital cameras (Microvision, Xi’an, China) with a maximum resolution of 1280 × 960 pixels. Each camera was equipped with a telephoto lens with a focal distance of 75 mm. Two LED (Light-Emitting Diode) light sources were used for lighting purposes. The image acquisition sensor adopted was a CCD (Charge-Coupled Device) solid-state image sensor (Microvision, Xi’an, China), and the imaging size could reach 6.4 mm × 4.8 mm. A PCI-E1394 acquisition card was adopted. With the application of an external signal trigger, the digital camera could continuously collect images, and the exposure range could be from 0.0001 to 30 s. In order to suppress the image saturation caused by thermal radiation under high temperatures, a band-pass filter was installed in front of the lens. This equipment used a 450 nm band-pass filter with a transmittance of 87.93%. A picture of this part is shown in Figure 3b. In the experiment, a program on the computer was used to control the start and end of the data collection and to set parameters for the data collection.

**Figure 4:**
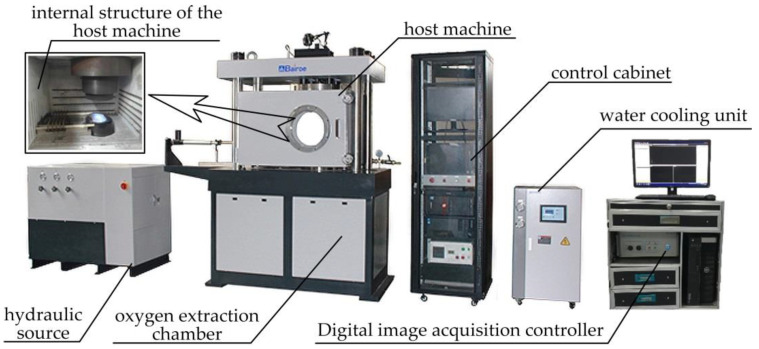
Overall diagram of the warm-forming-limit measurement equipment.

The overall diagram of the finished warm-forming-limit measurement equipment is shown in Figure 4.

### 2.3. Working Procedures

The measurement process of the warm-forming-limit measurement equipment in this paper is as follows:

1. Camera calibration: Adjust the direction of the two LED light sources to ensure that the light can pass through the glass above the host and that there is light on the specimen. Use the two cameras to shoot the calibration board from different directions to acquire images, identify the three-dimensional coordinates of the calibration points, and obtain the accurate inner parameters and external position parameters of the cameras through calculations, thus preparing for the acquisition of images in the experimental process.
2. Argon gas is injected through the air inlet to exhaust the oxygen so as to reach the preset values of pressure.
3. Heat the internal space environment of the chamber to the preset temperature and keep the temperature constant.
4. The specimen is pushed with the electric drive pusher to the preset position on the blank holder along the guide rail. The specimen gradually rises to the same temperature as the space environment inside the closed cabinet. If the specimen needs to be heated rapidly, according to the specific experimental parameters, it is quickly heated to the preset temperature with a rapid heating coil, and then moved along the guide rail to the preset position on the blank holder with the electric drive pusher.
5. Start the hydraulic system and control the displacement to make the blank holder move upward until the lifted specimen is under the plane joint with the die. The blank holder will then continuously and tightly press the specimen with the preset force. The punch will move upward according to the forming speed in the settings, and the bulging experiment process is started after making contact with the specimen. Serial images of the specimen will be collected over the whole forming process. If quenching is required for the specimen in the process, the water-cooling unit can be opened and the corresponding cooling rate can be set. In terms of experiments without any phase changes in the specimen, the water-cooling unit may also be started after the forming of the specimen to cool down the specimen and die in the heating furnace in order to facilitate the removal of the specimen.
6. Use the forming-limit measurement software developed to carry out procedures such as “define the computing area and conduct the digital speckle matching and computing”, “calculate the surface strain field”, and “generate cross section in the obtained strain field to acquire the limiting strain” to obtain the maximum and minimum strain limits of the specimen. The specific information can be found in our previously published literature [16].

## 3. Materials and Methods

### 3.1. Specimen

**Figure 5:**
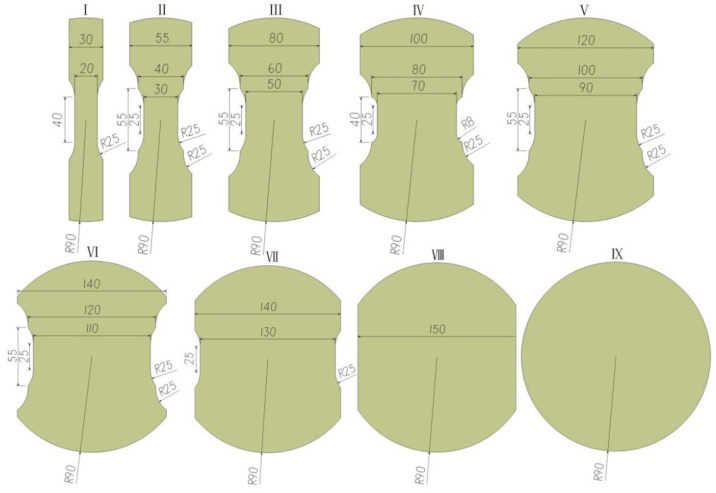
Schematic diagram of specimen size (unit: mm).

The equipment developed in this paper can be used to measure the FLC of DP600 with reference to the requirements in ISO/FDIS 12004-2-2008 [20], China’s national standards GB/T 15825.8-2008 [21] and GB/T 24171.1-2009 [22]. The thickness of the steel plate was 1.5 mm. In order to obtain the limiting strain data under the different strain paths of single-direction and dual-direction stretching, this research adopted specimens of outer arc-shaped sheet materials with nine different width dimensions. The outer diameter of all specimens was 180 mm, and the widths ranged from 20 to 180 mm. Refer to Figure 5 for the dimensions of these specimens.

The black and white high-temperature paint which was produced by Sherwin-Williams Company with the VHT brand was applied to produce random speckles on the surfaces of the specimens to help the computer to analyze and compute the specimen images collected. The speckle can withstand high temperatures of up to 704 °C with the high-temperature paint.

### 3.2. Warm-Forming and Room-Temperature-Forming Experiments

A hot-dip galvanizing coat was applied on the surface of the steel plate as lubrication in order to conduct the forming experiments on DP600 steel plates at 300 °C according to the procedure specified in Section 2.3. During the experiments, the specimen was heated to 300 °C with the internal space environmental heating coil of the cabinet body, and the water-cooling circulation was disabled. For the purpose of comparison, forming experiments were also carried out at room temperature (28 °C). The forming strain rate applied in the experiments was 0.01/s, the blank holder force was 150 KN, and the image collection frequency was 10 pieces/s. Three specimens of each width at the same temperature were used for the repeatability experiments.

### 3.3. Numerical Simulation

The DYNAFORM explicit dynamic analysis software (v5.7, Engineering Technology Associates, Inc., Detroit, MI, USA) was applied to simulate the forming process of the forming of DP600 at room temperature (28 °C) and 300 °C. The material adopted was in a square No. 16 fully integrated shell unit grid with five integral points in the thickness direction. The material model adopted was 36*MAT_3-PARAMETER_BARLAT, which is a kind of elastic–plastic model suitable for sheet metal forming analysis. The Young’s modulus was 207 GPa, the density was 7.83 × 10^−6^ kg/mm^3^, Poisson’s ratio was 0.3, and the plastic strain ratio was 0.812. To obtain better calculation precision, the size of the grid was set to 1mm, the die was set with a rigid body, the strain rate of the sheet material was 0.01/s, the blank holder force was 150 KN, and the friction coefficient was 0.15. In terms of the numerical simulation process for the forming temperature of 300 °C, the temperatures of both the sheet material and the various forming dies were kept constant at 300 °C. Considering that the micro-structure of DP600 will not incur any obvious changes at the temperature of 300 °C [23], the numerical simulation process adopted the true stress–strain curve parameters when the material was at the temperature of 300 °C, without considering the environmental heat radiation and thermal conduction among the dies and sheet materials. The stress–strain relationship of the DP600 material at room temperature and 300 °C was taken from data in the literature [24].

**Figure 6:**
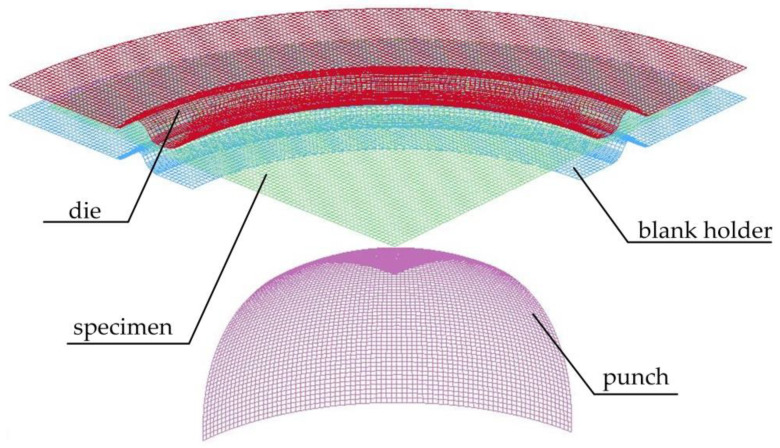
Numerical simulation model diagram.

The finite element model is shown in Figure 6. Since the sheet material was in a symmetrical shape, a 1/4 model was adopted for the simulation. The motion parameters of the various components were consistent with those in the experiment, which meant that the blank holder would first move upward through the stroke control, and then clamp the sheet material together with the die. The blank holder was then switched to the control of the constant blank holder force, and the punch started moving upward through the stroke control. Different from the control method of equipment, in the DYNAFORM software (v5.7, Engineering Technology Associates, Inc., Detroit, MI, USA), it was impossible to set the experiment to automatically stop when the forming force dropped by 5%. In order to achieve the numerical simulation process of full deformation and fracture of the sheet, the punch was controlled by stroke of the sheet metal cracked in the experiment plus 10 mm. The fracture criterion is included in the material model of 36*MAT_3-PARAMETER_BARLAT of DYNAFORM software. The fracture criterion is used to calculate the limit strain by calculating thickness *t* and hardening exponent *n* in the material model.

## 4. Results and Discussion

### 4.1. Experimental Results

**Figure 7:**
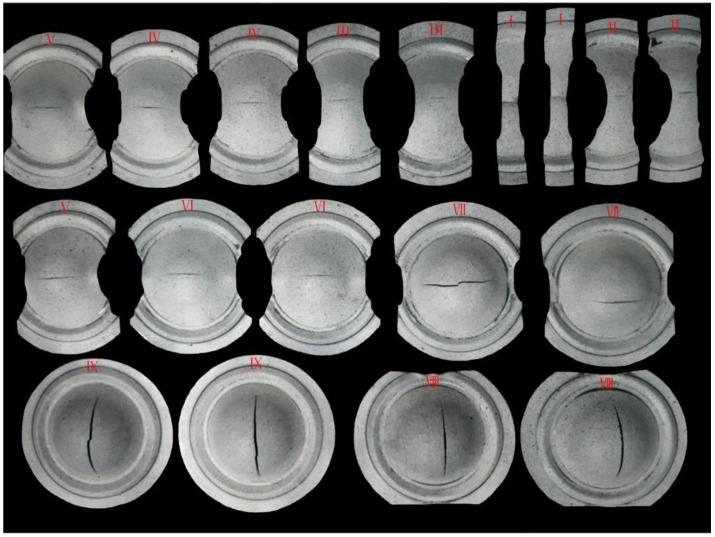
Specimen obtained from the experiment at the temperature of 300 °C.

**Figure 8:**
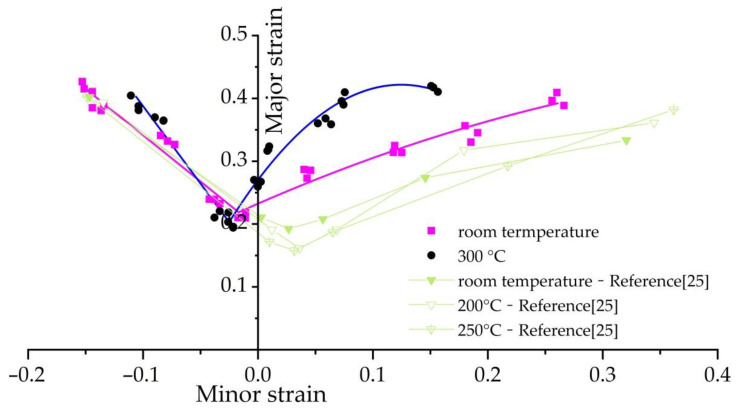
Forming limit curve diagram of DP600 steel at room temperature and 300 °C in this paper. The line is fitted to obtain the average of the measurement results for three samples of each width. The results from the literature [25] are also shown.

The shape of the specimen after the stamping and forming at 300 °C is shown in Figure 7. All crack directions were nearly perpendicular to the long axis of symmetry of the specimen. The forming limit diagram obtained and compared with the literature is shown in Figure 8.

It can be seen in Figure 8 that the forming limit of DP600 steel at 300 °C was generally higher than the forming limits of the material at room temperature (28 °C). The reason for this phenomenon is that the thermal activation of dislocations makes the plastic deformation of high-strength steel easier with the increase in material temperature [26]. In terms of the influence of temperature on the forming limits, the values of major strain had relatively small differences from those at room temperature in the left area, when the minor strain was less than 0, while the values of the major strain were obviously higher than the values at room temperature. In other words, the limiting strain of DP600 steel under tension–tension strain status suffered a significant influence from temperature, while the limits under tension–compression strain status suffered an insignificant influence from temperature with the same trend reflected in the literature [26]. The results were also consistent with the law of the influence of strain status on the forming limits of metal that was discovered in the literature [27]. The forming limits of DP600 steel obtained in this paper at 300 °C were lower than the forming limit at a normal temperature with the minimum principal strain in the interval [−0.06, −0.02], but the forming limits outside the interval [−0.06, −0.02] were all higher than those at a normal temperature in other minimum-principal-strain intervals. The reason for this phenomenon is that the blue brittleness of carbon steel and dynamic strain aging (DSA; can be described as an unexpected strengthening in the flow stress in a specific temperature range) work together. On the one hand, the blue brittleness of carbon steel easily appears in the range of 200–400 °C [28], which reduces the toughness and plasticity of the material, as well as the forming limit. On the other hand, the strain aging phenomenon will occur with the deformation of the material [26], and the strain aging effect of steel is the strongest at around 300 °C; the strain aging effect is significant when the strain is high [29]. It should be noted that the major strain in the literature [26] was generally lower than that obtained in this paper. The main reason is that the strain rate in the literature [26] is higher than that of the experiment in this paper [30]. The above results demonstrated that the warm-forming limit measurement equipment designed and manufactured in this paper provided a rather rapid and accurate solution for the measurement of warm-forming limits of high-strength steel.

### 4.2. Numerical Simulation Results

**Figure 9:**
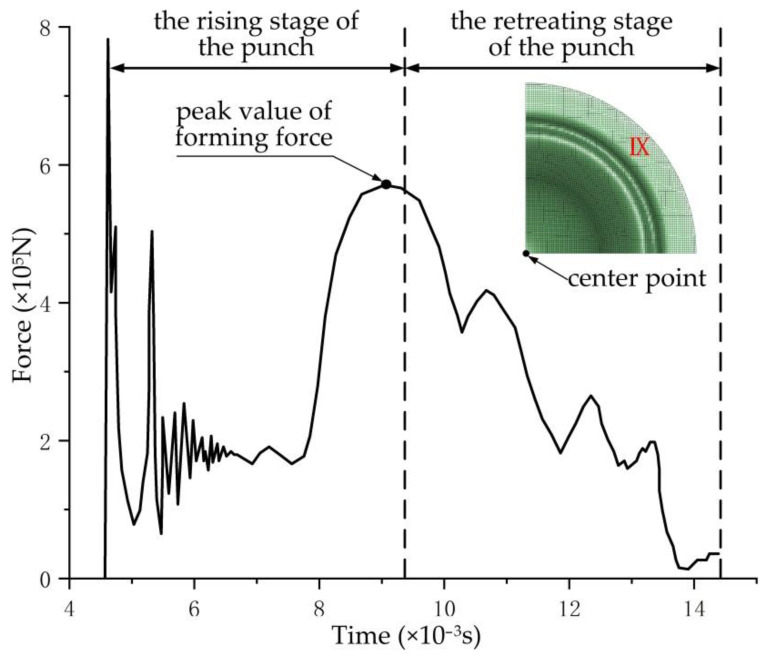
Curve for the contacting force of the center point between the punch and sheet in the simulation process of specimen IX at 300 °C.

After the completion of the forming simulation of DP600 steel at 300 °C and at room temperature, taking the specimen IX at 300 °C as an example, the curve for the contact force of the center point between the punch and sheet was extracted at the time of the maximum force of the punch with the judgment method [19] as shown in Figure 9. When the punch was in contact with the sheet material, the forming force (Y value) reached the peak value. In the subsequent process of stabilized forming, the forming force would reach the peak value again. As the forming continued, the grids in the most severe forming area would encounter distortion due to excessive stretching, leading to a gradual decline in the contact force between the punch and the sheet material. The time when the contact force declined to 5% of the peak was selected as the time for the calculation of the limit strain.

**Figure 10:**
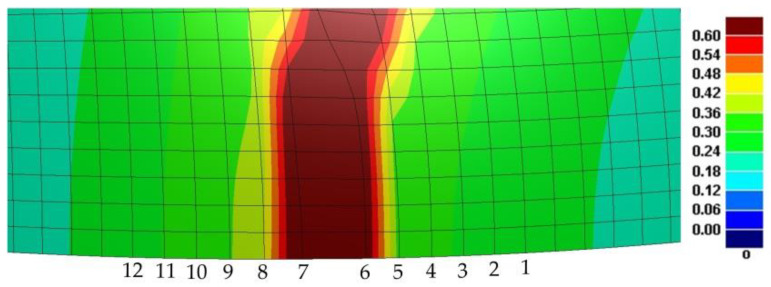
Distorted grid formed at the fracture point in the numerical simulation of specimen IX at 300 °C. The values shown in the figure are the maximum principal strain.

The data on the changes in strain and shapes of the grid of the sheet material at each time point—starting from the occurrence of the deformation to the occurrence of rapid distortion—could be obtained from the simulation. In the practical experiment’s process of stamping and forming, the material on both sides of the fracture would not be connected after the fracture of the sheet material, and the deformations on both sides of the fracture would not come across any changes after the release of the stress. Therefore, this measurement could be used to obtain the strain status after the fracture. In the numerical simulation with the DYNAFORM software, however, the grid would not fracture after reaching the fracture time of the sheet material [17]. Instead, the grid would be further extended to become a distorted grid, taking the simulation of specimen IX at 300 °C as an example, as shown in Figure 10, which is completely different from the actual fracture of the material at the place of the crack. Therefore, the strain values at the nearest nodes on both sides of the cracked grid were inaccurate. Therefore, this paper proposes that the strain values of the nodes on both sides of the grid with distortion could be removed in the process of acquiring the strain values of the fracture limits of the sheet material. The limit strain grid for the specimen could be identified and analyzed in each width with the judgment approach for the moment of occurrence of the maximum punch force, and the limit strain can be calculated. 

**Figure 11:**
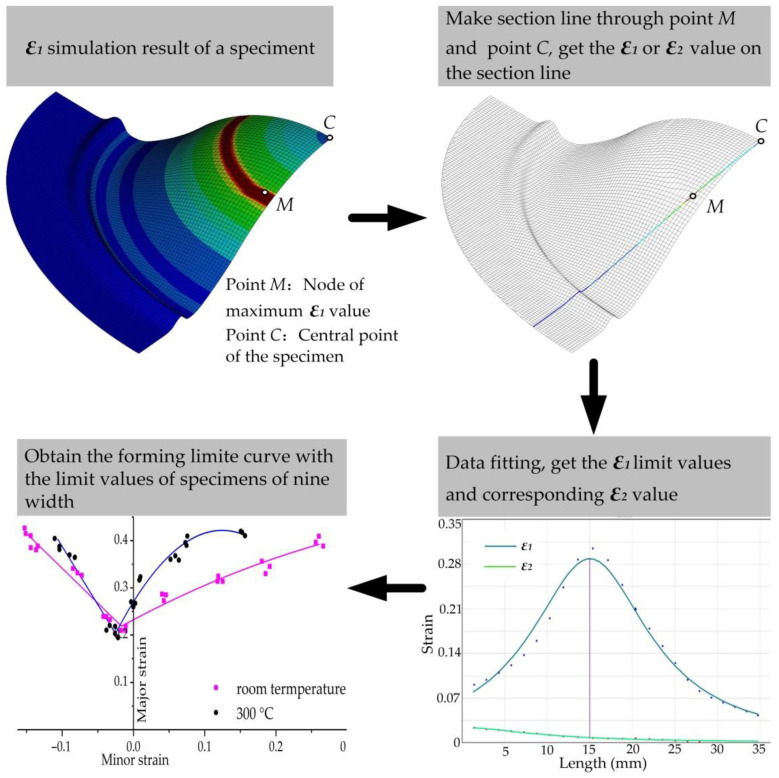
Schematic diagram of calculating the forming limit values.

A schematic diagram of calculating the forming limit values from the simulation result is shown in Figure 11. 

The specific process is explained below:

1. Select the ultimate strain moment of the grid, find the point where the maximum principal strain is located, connect it with the central point of the specimen to make a cross line, and obtain the maximum principal strain $\varepsilon_{1}$ and minimum principal strain $\varepsilon_{2}$ of the node on each grid of the cross line, as well as the length of the arc line from each node to the central point of the specimen. The above values should then be imported into the quadratic function polynomial $f\left(x\right)={\mathrm{ax}}^{2}+\mathrm{bx}+c$, where x represents the length of the arc line from the strain measurement node to the center of the specimen, and f(x)  represents the value of the maximum principal strain, $\varepsilon_{1}$ or $\varepsilon_{2}$. This method is adopted to select and obtain the $\varepsilon_{1}$ or $\varepsilon_{2}$ values of m nodes on both sides of the distorted grid, except for the No. 6 and No. 7 nodes (refer to Figure 10). The values of a total of 2 m nodes are obtained (m = 2,3,4,5…, and the number of nodes taken is symmetrically distributed on both sides of the distorted grid).
2. In the smooth data area, the quadratic parabolic function of any 2 m continuous strain measurement points $x_{1}$, $f_{1}\left(x\right),{\;x}_{2}$, $f_{2}\left(x\right)$……, ${\;x}_{2n}$, $f_{2n}\left(x\right)$ on the cross-sectional line is obtained by interpolation.
3. Use $f\left(x\right)={\mathrm{ax}}^{2}+\mathrm{bx}+c\;$ to perform least squares fitting for 2 m consecutive points on the same cross-sectional line to obtain the corresponding extreme value of f(x), which is the limit value of the major strain, and determine the value of x’ for x that corresponds with the time when the value is obtained. Apply the same method to perform quadratic function fitting for $\varepsilon_{2}$, and take the $\varepsilon_{2}$ that corresponds with the value of x’ as the limit value of minor strain.
4. Use the above method to calculate the forming limit values of the specimens at each width to obtain the forming limits under nine width conditions.

**Figure 12:**
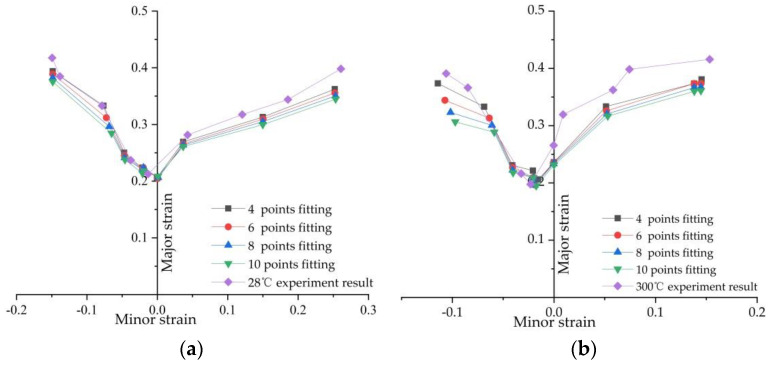
Comparison between the calculation results of the forming limit curve for different numbers of fitting points and the experimental results at room temperature 28 °C (**a**) and at 300 °C (**b**).

**Figure A1:**
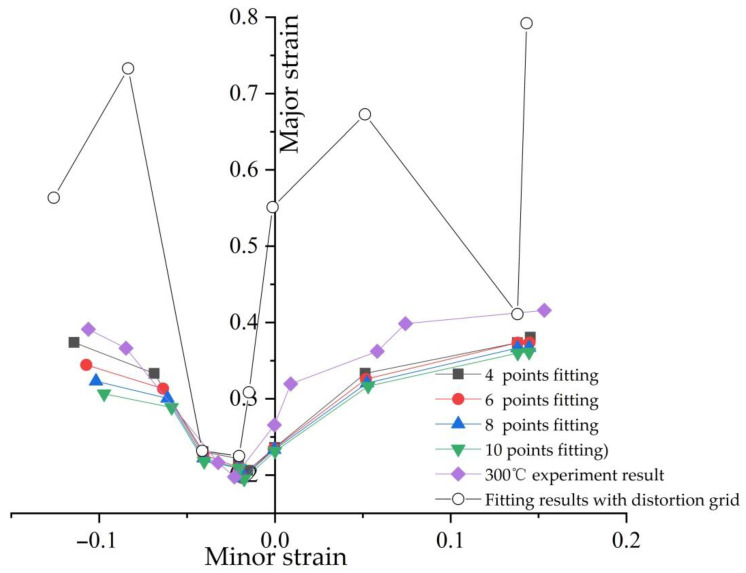
Comparison between the calculation results of the forming limit curves for different numbers of fitting points and the experimental results at 300 °C with a distortion grid.

In accordance with the fitting method for the forming limits of sheet materials, remove one node from each of the two sides of the distorted grid, and perform fitting with 2 m nodes on each side. To illustrate the influence of the number of nodes participating in the fitting on the fitting results of the forming limit curve, m = 2, 3, 4, 5 were selected for the fitting, and a comparison diagram was obtained for the forming limit results and the experimental results at the temperature of 300 °C and room temperature (28 °C), as shown in Figure 12. It can be seen from the diagram that the forming limit curve obtained from the previous method had a consistent change trend with the forming limit curve obtained in the experiment, regardless of the values selected for m; this was obviously better than the calculation and fitting results obtained without removing the distorted grid (refer to Figure A1 in the Appendix A). The results demonstrated that, after removing distorted grids, the acquisition and fitting method for the forming limit curve proposed in this paper, which is based on the numerical simulation results obtained with the DYNAFORM software, is feasible. It can also be seen in the figure that the lower the selected value of m is, the closer the forming limit curve calculated in the simulated calculations will be to that of the experimental results under both temperature conditions. A possible reason is that in the process of calculating the limit strain of each width sample, the section line perpendicular to the crack direction is made and the strain value is taken on the section line. When the m value is smaller—that is, the closer the strain value is to the crack location—the material is more affected by necking and fracture behavior during crack formation, which can better reflect the limit strain at fracture, so that the fitting results are closer to the experimental values.

## 5. Conclusions

This paper researched and developed a set of measurement equipment for the warm-forming limits of sheet materials based on the Nakazima bulging test method and digital image correlation method. The equipment was able to achieve non-contact measurement for the forming limits and strain of steel under multiple temperature conditions. The equipment was applied to measure the forming limits of DP600 at 300 °C, and a forming limit diagram was established. The performance of the equipment was verified through a comparison with the results in the literature. The experimental results show that the effect of temperature on the forming limit of DP600 steel is related to the strain state.

A numerical simulation of the bulging test was carried out with an application of the DYNAFORM software, and a limit-strain-fitting method was proposed to eliminate the influence of distorted grids. The comparison with the experimental results verified the accuracy of this fitting method. The results show that when the method proposed in this paper is used to fit, the lower the number of nodes involved in the fitting is, the closer the forming limit curve is to the experimental results.

## Figures and Tables

**Table 1:** Main performance parameters of the warm-forming part.

Indicator	Parameter
Maximum stamping force (KN)	600
Forming speed (mm/min)	0–750
Maximum clamping force (KN)	600
Stamping stroke (mm)	0–150
Sheet material thickness range (mm)	0.2~4.0
Sheet material width range (mm)	0~220
Specimen heating temperature (°C)	Up to 900
Cupping punch diameter (mm)	100

## Data Availability

The data supporting reported results are available on request from the corresponding author.

## Abbreviations

Abbreviations, acronyms, and symbols	
FLC	Forming limit curve
FLD	Forming limit diagram
PLC	Programmable logic controller
LED	Light-emitting diode
CCD	Charge-coupled device
ε_1_	Maximum principal strain
ε_2_	Minimum principal strain
m	The number of nodes
n	Hardening exponent
t	Thickness
DSA	Dynamic strain aging
